# Supplementary figures and images for: Local vulnerability and global connectivity jointly shape neurodegenerative disease propagation
Source: PLoS Biol. 2019 Nov 21;17(11):e3000495. doi: 10.1371/journal.pbio.3000495 (PMC6894889; doi:10.1371/journal.pbio.3000495)

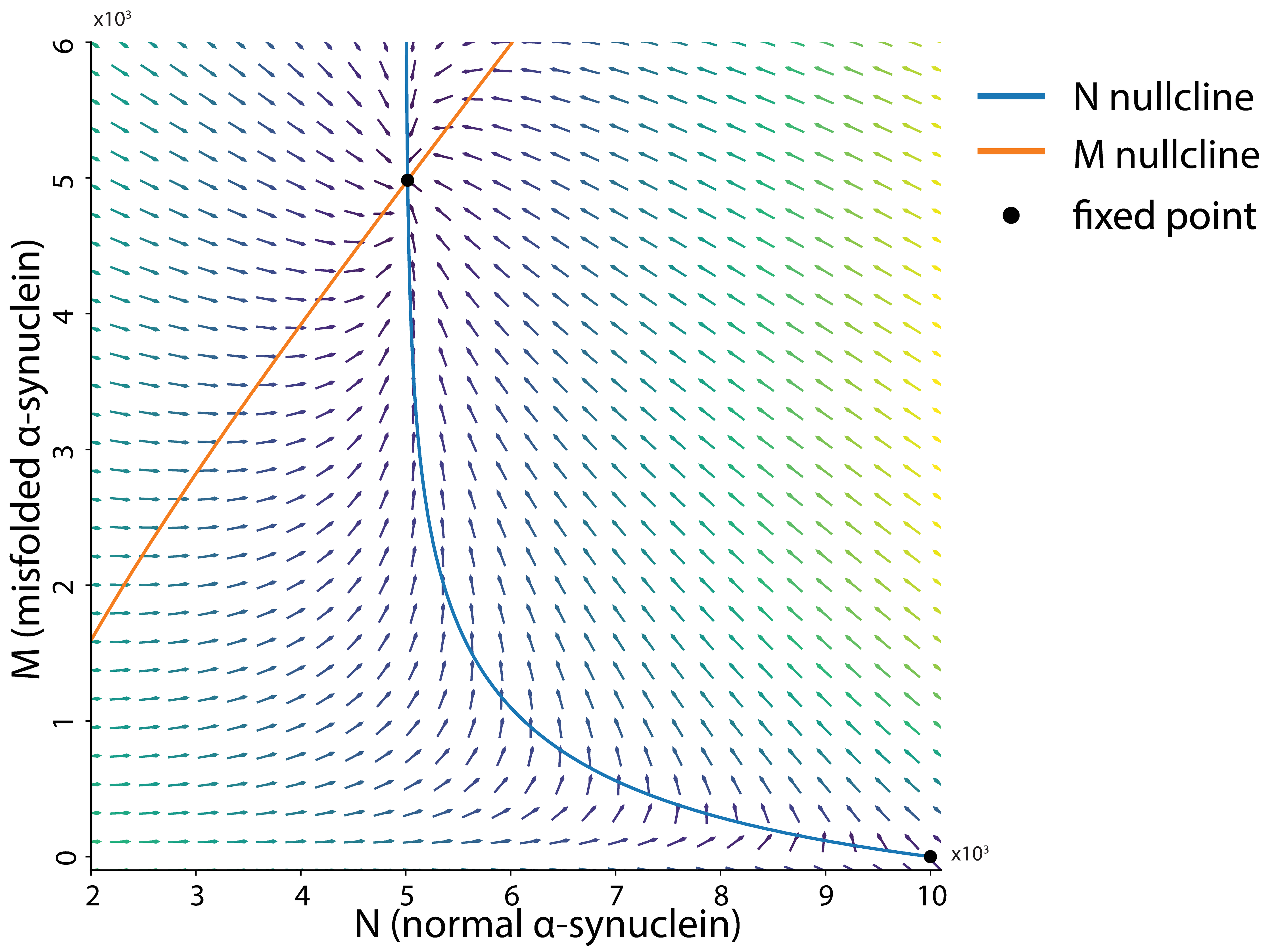

Supplement: S1 Fig — M decreases with N (N nullcline, blue, equation [S4]) and N increases with M (M nullcline, orange, equation [S5]), therefore, apart from (N = 10000, M = 0), there is only one other intersection (N = 5017.15, M = 4982.85) of the 2 lines, indicating that the system has 2 fixed points only. The vector field (arrows) denotes the direction of the gradient at each position (i.e., the system at that point will move along the direction of the corresponding arrow). The code to generate the figure can be found at https://github.com/yingqiuz/SIR_simulator/tree/master/results/S1_Fig.ipynb. (TIF) [file pbio.3000495.s003.tif]

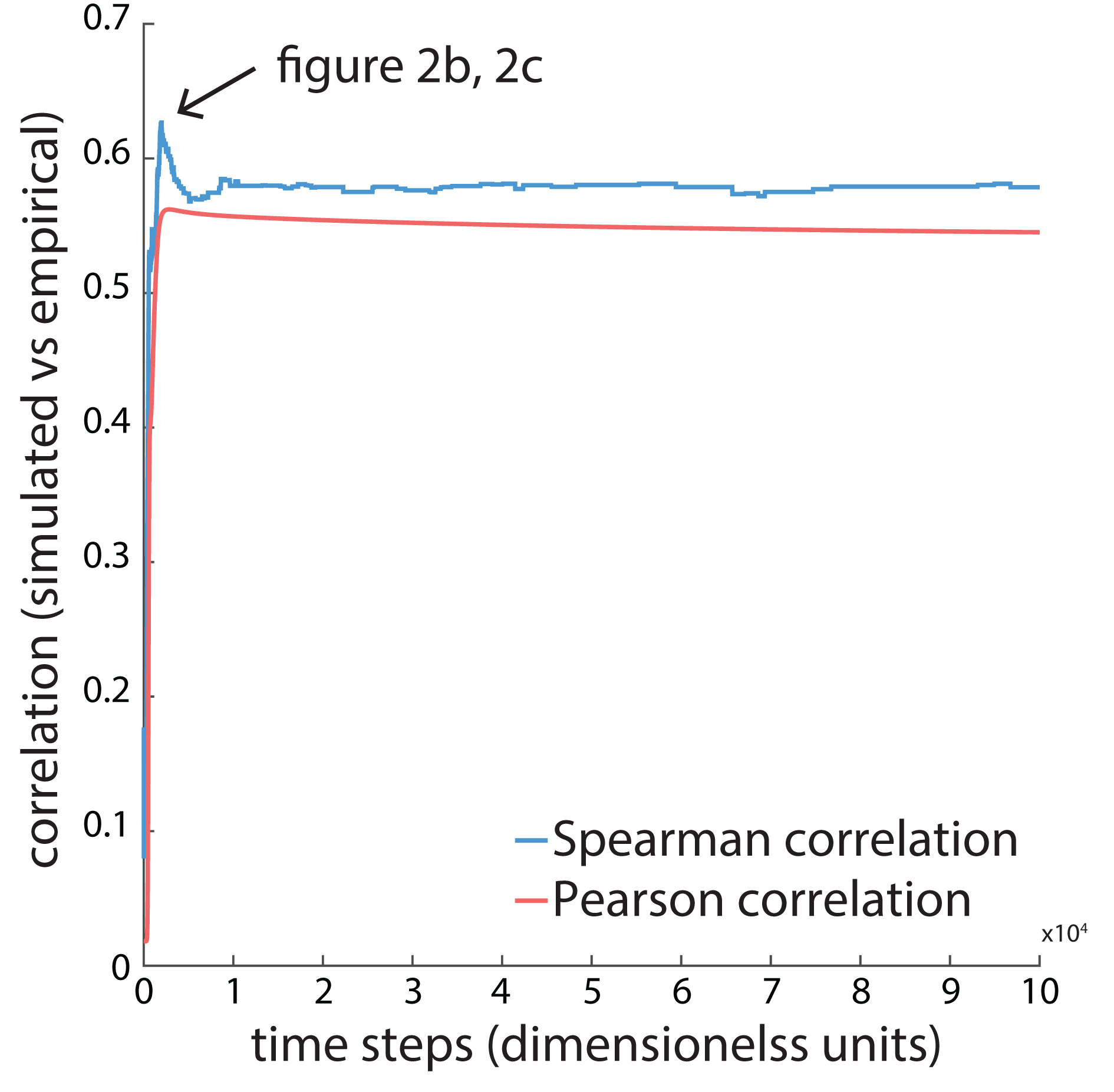

Supplement: S2 Fig — Correlations between simulated atrophy and empirical atrophy derived from PD patient DBM maps. Correlations are shown as a function of simulation time. At large t, the model fit stabilizes as the system approaches the stable point. The underlying data can be found at https://github.com/yingqiuz/SIR_simulator/tree/master/results/S2_Fig.mat. (TIF) [file pbio.3000495.s004.tif]

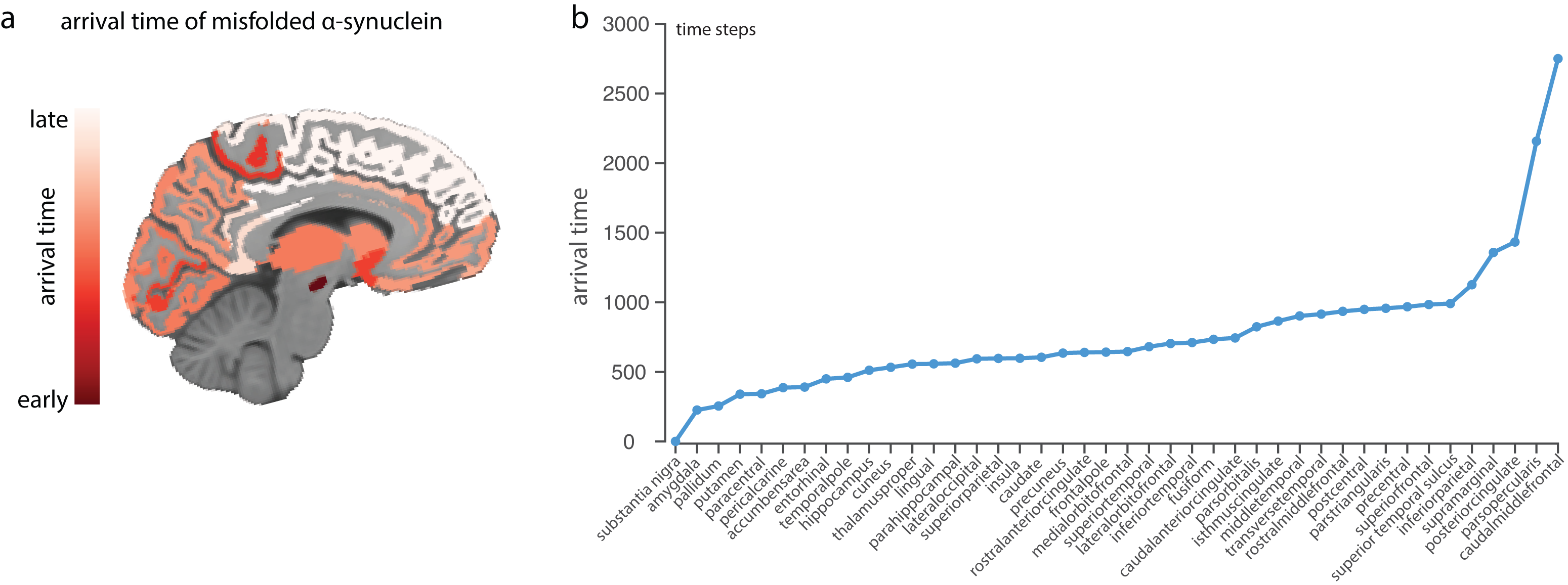

Supplement: S3 Fig — (A) Regional arrival time of misfolded α-synuclein is defined as the time steps required for misfolded α-synuclein amount to exceed 1 (after seeding at the substantia nigra with one misfolded agent). This roughly follows the Braak staging hypothesis (see [31]). (B) Arrival time of misfolded α-synuclein at each brain region. The underlying data can be found at https://github.com/yingqiuz/SIR_simulator/tree/master/results/S3_Fig.mat. (TIF) [file pbio.3000495.s005.tif]

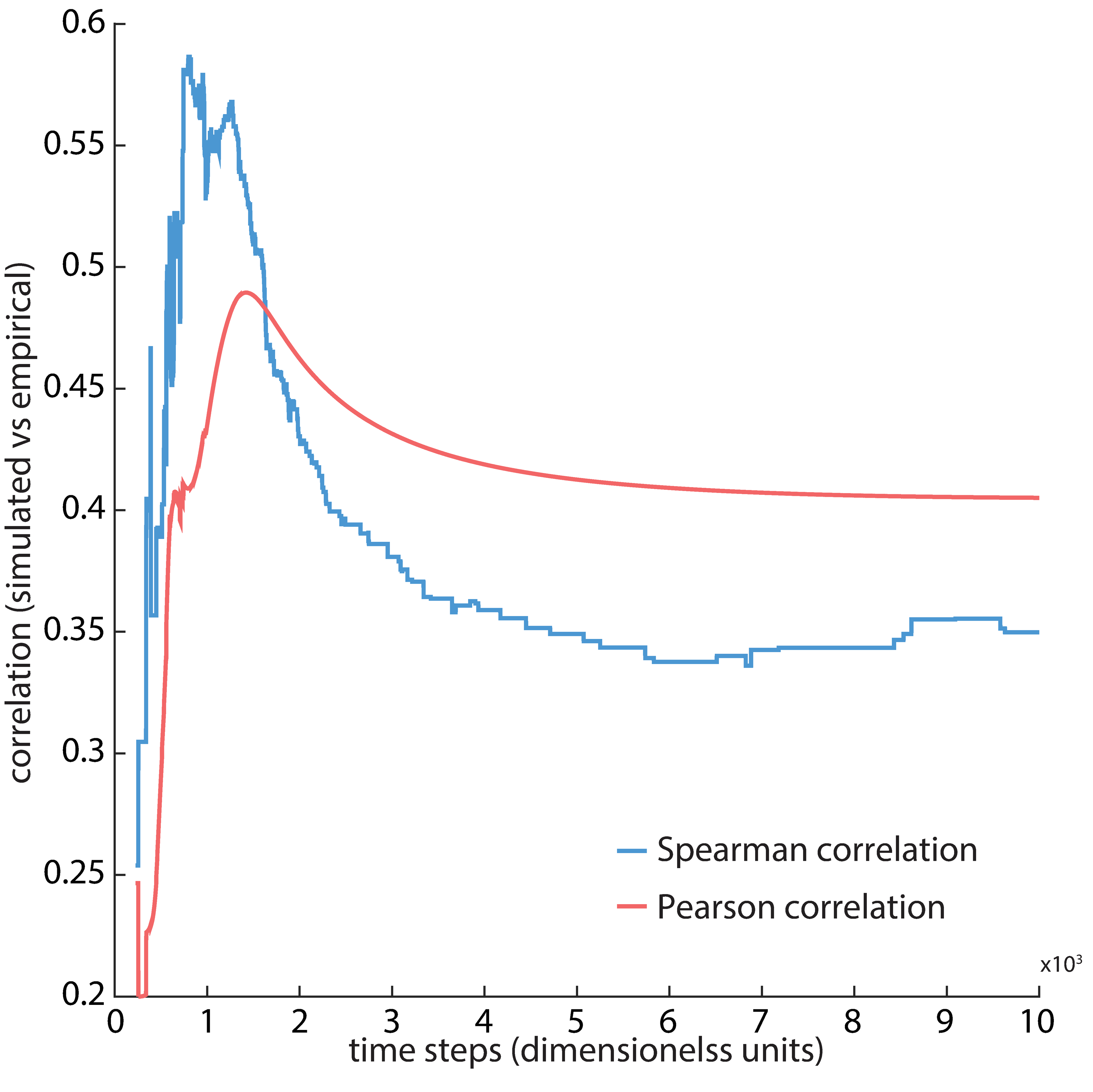

Supplement: S4 Fig — The computational model replicates empirical atrophy patterns estimated using an alternative DBM pipeline. Model fits are comparable between the minctools and FSL-estimated atrophy patterns. The underlying data can be found at https://github.com/yingqiuz/SIR_simulator/tree/master/results/S4_Fig.mat. (TIF) [file pbio.3000495.s006.tif]

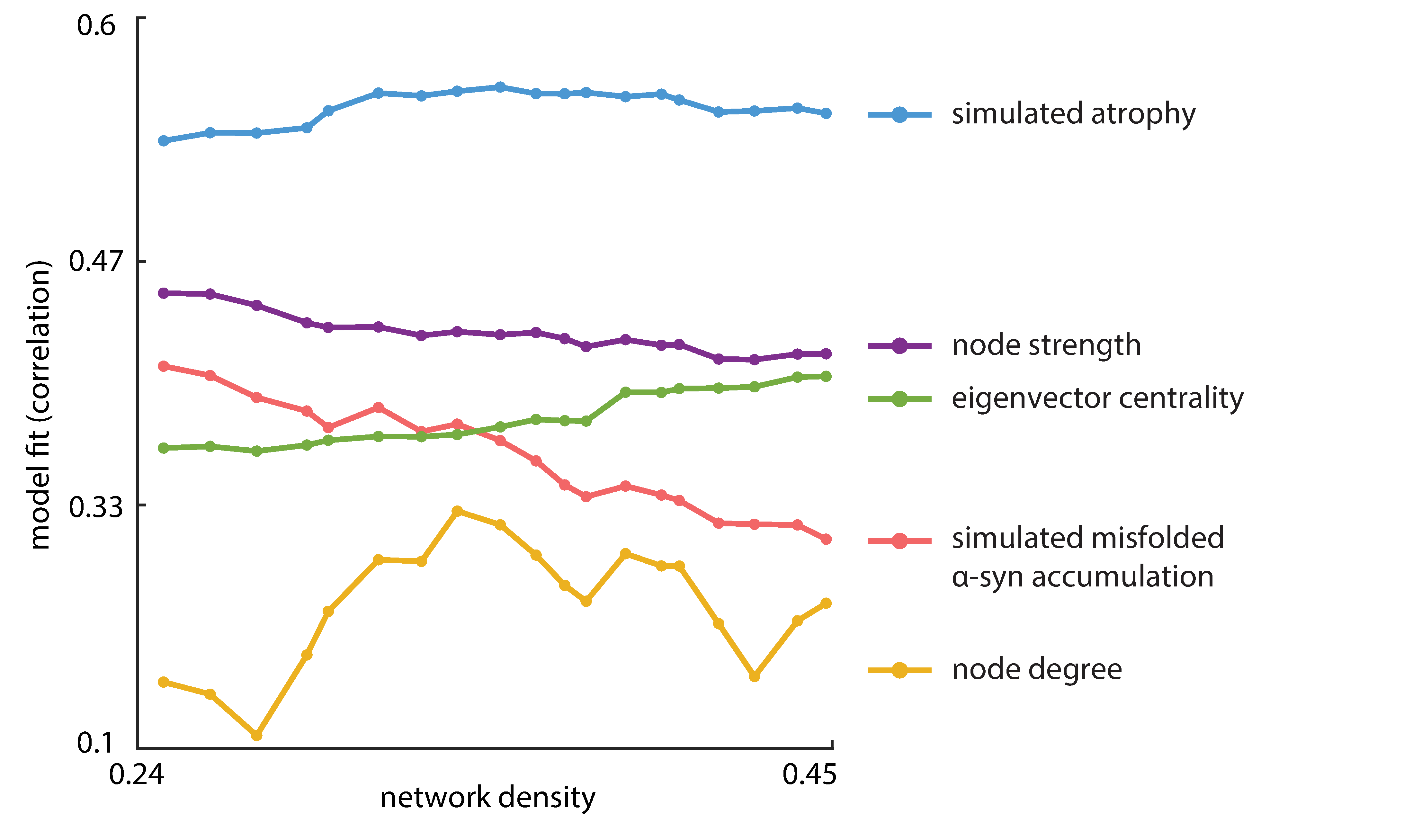

Supplement: S5 Fig — The model integrated with gene expression levels has more predictive power than the density of misfolded α-synuclein (red) and the static network metrics, including node degree (yellow), node strength (green), or eigenvector (purple) centrality. The underlying data can be found at https://github.com/yingqiuz/SIR_simulator/tree/master/results/S5_Fig.mat. (TIF) [file pbio.3000495.s007.tif]

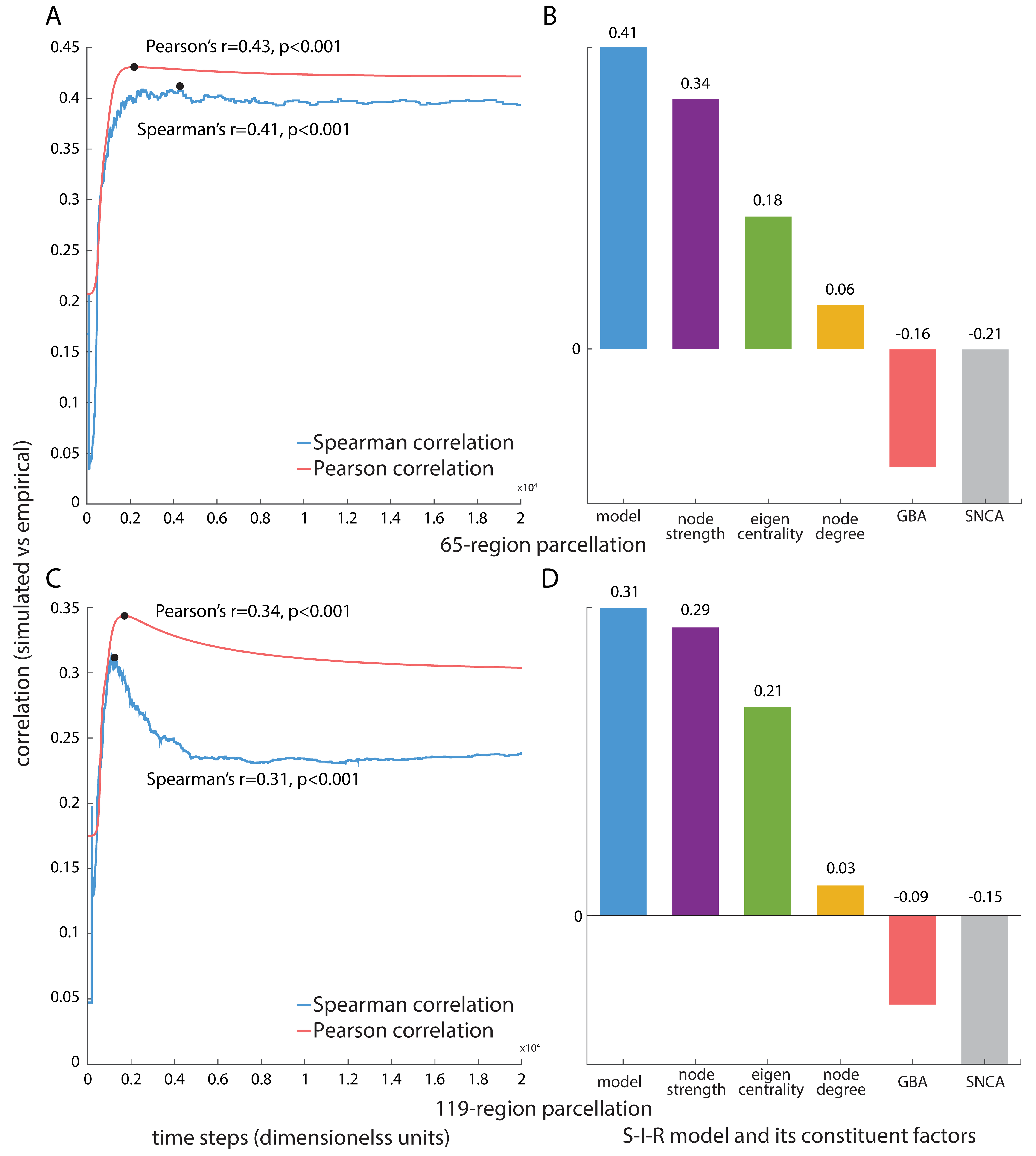

Supplement: S6 Fig — The 42 regions used in the main manuscript were hierarchically partitioned into 65 regions and then a further 119 regions. Simulations were conducted on these two finer resolutions, and yielded comparable results to the model fit at 42-region resolution. (A) Spearman’s correlation (blue curve) and Pearson’s correlation (red curve) versus time using the 65-region parcellation. Black dot: peak position of the correlation coefficients. (B) The model has more predictive power than its constituent factors (as assessed by Spearman’s correlation). (C) Spearman’s correlation (blue curve) and Pearson’s correlation (red curve) versus time using the 119-region parcellation. Black dot: peak position of the correlation coefficients. (D) The model has more predictive power than its constituent factors (as assessed by Spearman’s correlation). The underlying data can be found at https://github.com/yingqiuz/SIR_simulator/tree/master/results/S6_Fig.mat. (TIF) [file pbio.3000495.s008.tif]

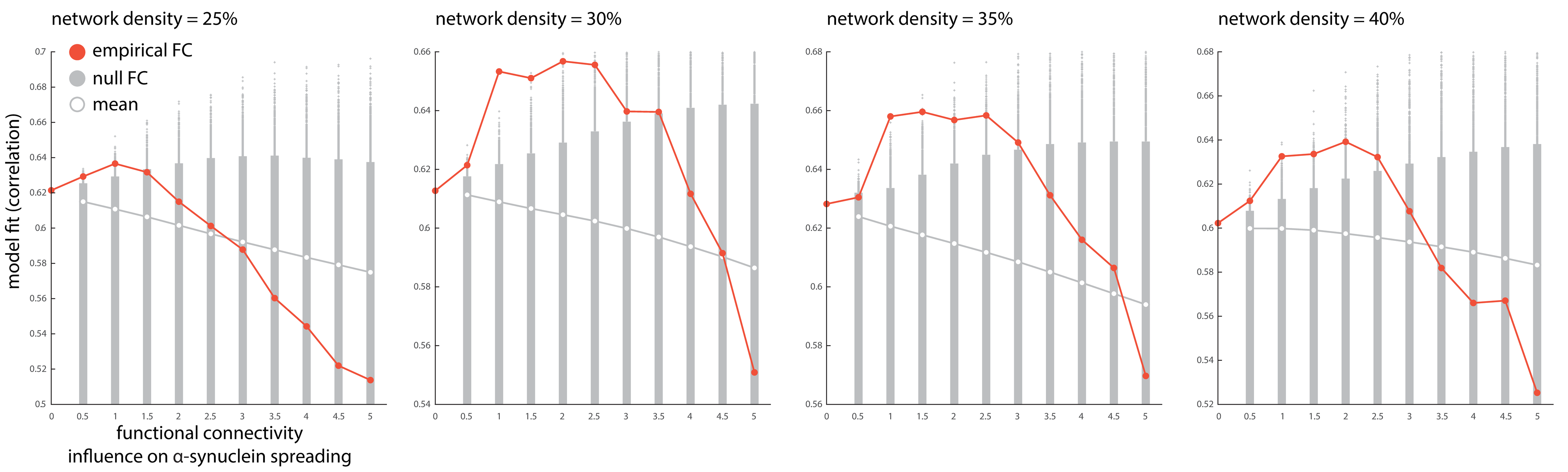

Supplement: S7 Fig — Increasing k (the influence of FC on α-synuclein transmission) first facilitates then degrades model fit. The red line indicates model fit using true FC values. For each k, resting-state fMRI time series were reassigned to construct null FC matrices. The null model fit declines monotonously as k increases (gray line). At smaller vaues of k, simulations based on real FC yield significantly higher model fit than the null settings as indicated by the 95% confidence interval (gray bar), whereas at larger k, real FC ceases to have advantage over null FC. The underlying data can be found at https://github.com/yingqiuz/SIR_simulator/tree/master/results/S7_Fig.mat. FC, functional connectivity. (TIF) [file pbio.3000495.s009.tif]

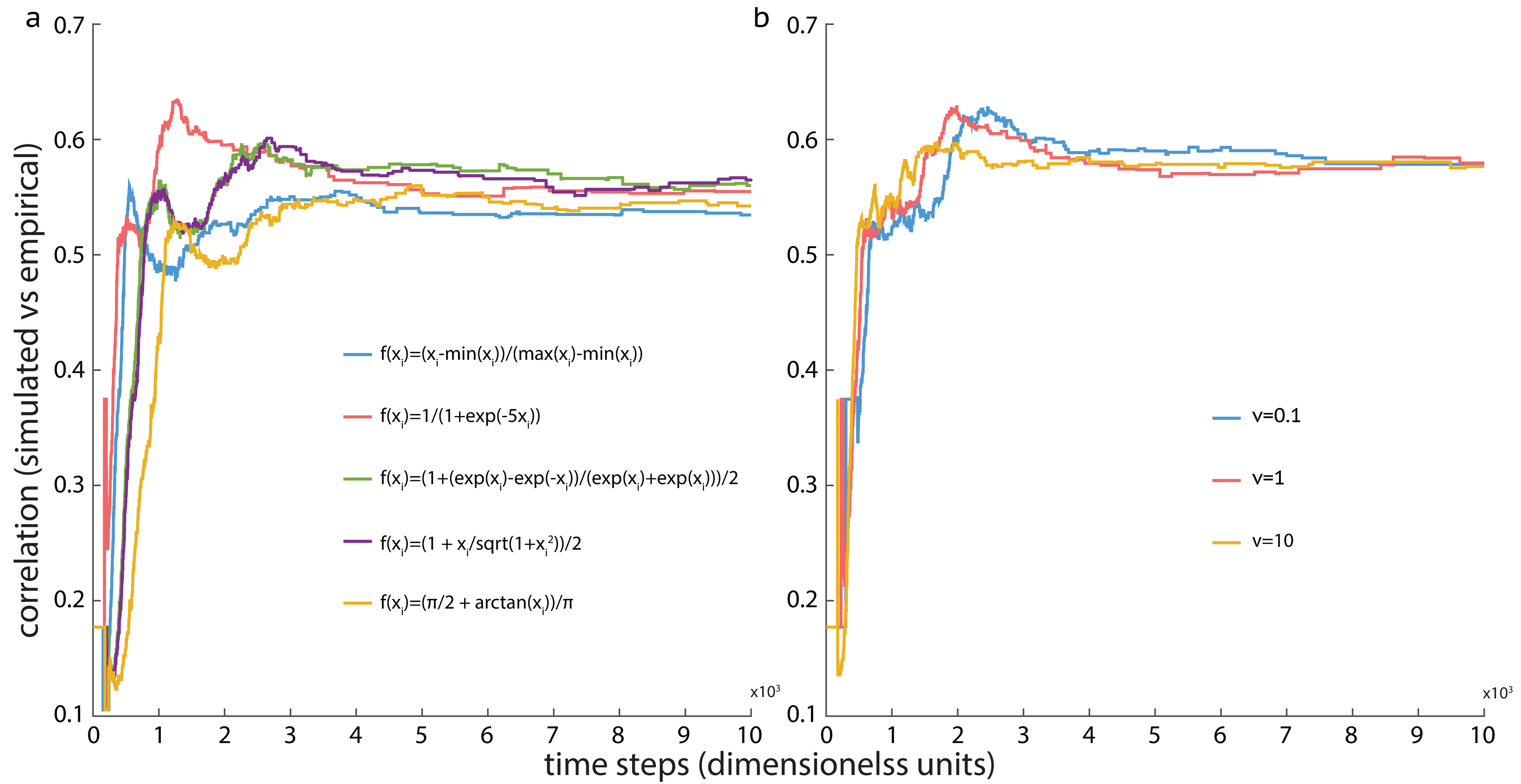

Supplement: S8 Fig — (A) In the main setting, we transformed the SNCA and GBA expression z-scores to synthesis and clearance rates using a standard normal cumulative distribution function (Fig 2). To test other possibilities of the relation between gene expression and synthesis or clearance rate, we chose a set of commonly used functions that have domain of all real numbers and return values monotonically from 0 to 1. The temporal pattern of model fit is robust to the choice of transformation functions: the model yields similar results as long as the relative magnitudes of regional gene expressions are preserved. xi is the gene expression z-score in region i and f(xi) is the transformation function. (B) We set the probability of exiting an edge (i,j) to the reciprocal of edge length li,j in the main setting. However, it is possible that the protein agents propagate faster or slower than the regional synthesis or clearance process. To test sensitivity of the model to the rate of protein propagation, we introduced propagation speed v and set the probability thereof to v/li,j such that varying v changes the relative scale of the propagation process vis-a-vis the regional synthesis and clearance process (e.g., increasing v suggests that the propagation process happens faster than the regional synthesis and clearance processes). We chose v = 0.1,1,10 (where v = 1 corresponds to the results in the main text) and found that the relative scale of the two processes has little effect on the model fit. https://github.com/yingqiuz/SIR_simulator/tree/master/results/S8_Fig.mat. (TIF) [file pbio.3000495.s010.tif]

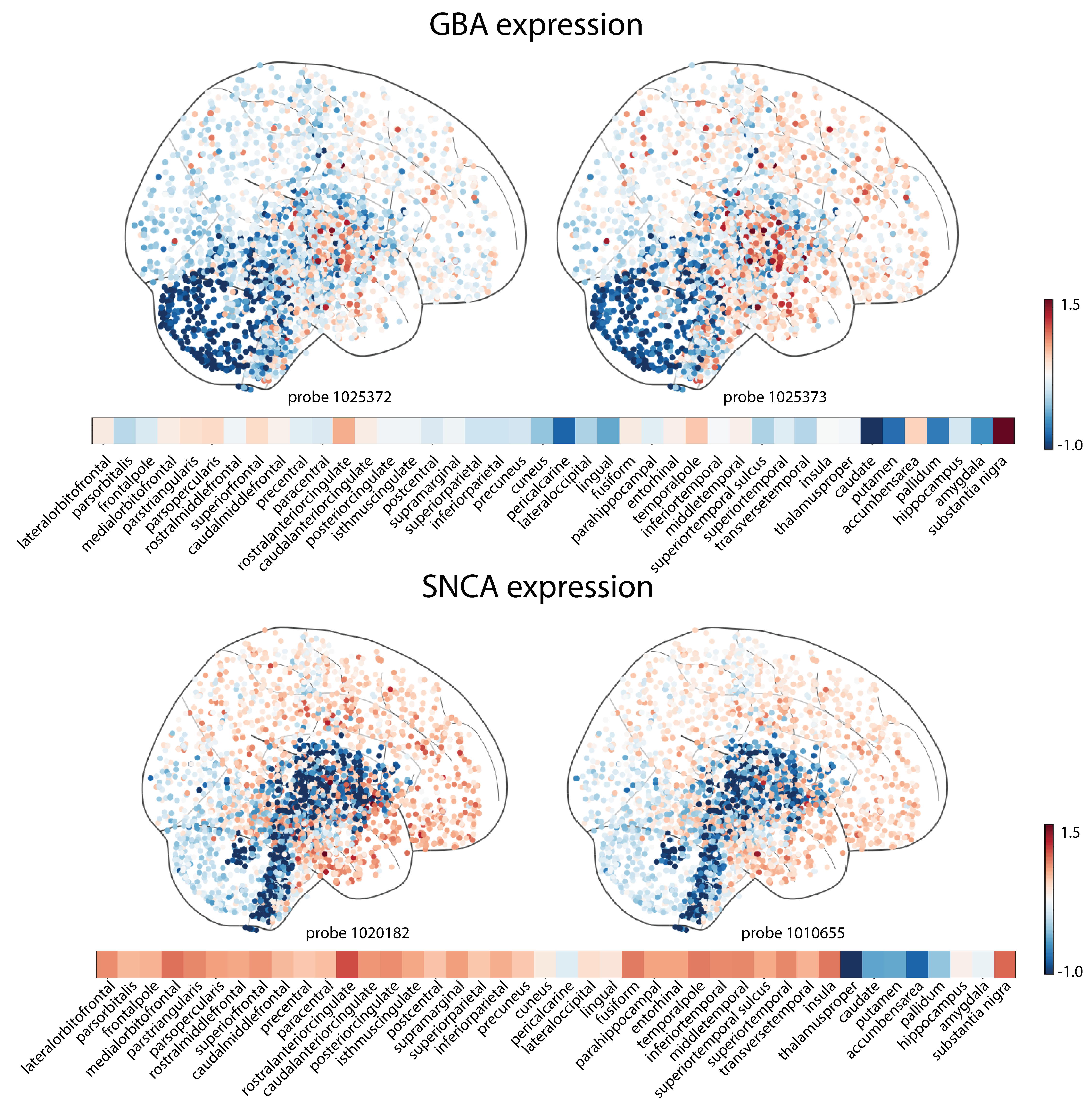

Supplement: S9 Fig — (A) Regional GBA expression. There are 3 probes for GBA (probe ID: 1025372, 1025373, and 1025374). Probes 1025372 and 1025373 were included to generate the group transcription profile. Probe 1025374 was excluded as it deviated too much from probe 1025372 (Pearson correlation = 0.30), while the correlation between the other two probes is 0.79. (B) Regional SNCA expression. Probes 1020182 and 1010655 were included to generate the group transcription map. Compared to GBA expression, SNCA is more homogeneous in cortical regions. The underlying data can be found at https://github.com/yingqiuz/SIR_simulator/tree/master/results/S9_Fig.mat. (TIF) [file pbio.3000495.s011.tif]

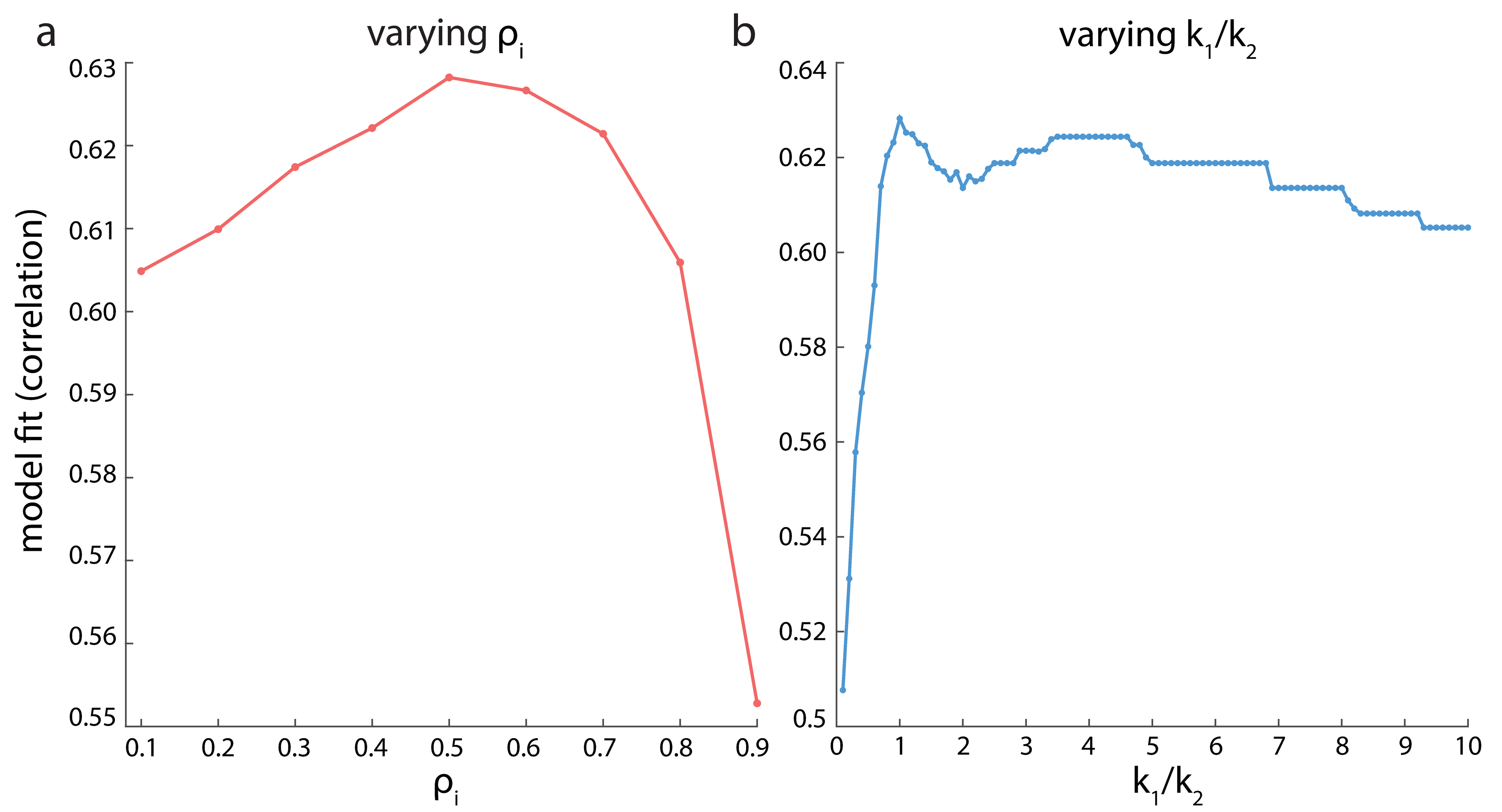

Supplement: S10 Fig — Model fit (Spearman’s correlation) is robust to variations in ρi, k1, k2 (results shown at network density 35%). (A) ρi controls the probability of remaining in region i while (1-ρi) is the probability of exiting region i per unit time. The main results are based on ρi = 0.5. However, the model fit is consistently above 0.55 across ρi ranging from 0.1 to 0.9. (B) For the atrophy in region i, k1 controls the contribution of α-synuclein accumulation inside region i, while k2 controls the contribution of deafferentation induced by atrophy in connected regions. k1 + k2 = 1. The model fit is consistently over 0.5 across k1/k2 ranging from 0.1 to 10. These results suggest that the predicative power of the model is robust to variations in free parameters ρi or k1/k2. The underlying data can be found at https://github.com/yingqiuz/SIR_simulator/tree/master/results/S10_Fig.mat. (TIF) [file pbio.3000495.s012.tif]
