# Supplementary material for: Local vulnerability and global connectivity jointly shape neurodegenerative disease propagation
Source: PLoS Biol. 2019 Nov 21;17(11):e3000495. doi: 10.1371/journal.pbio.3000495 (PMC6894889; doi:10.1371/journal.pbio.3000495)
Supplement: Attachment — Submitted filename: PLoS Biology Replies to Reviewers.docx [file pbio.3000495.s013.docx]

Dear Editor(s) and Reviewers,

Thank you for the encouraging comments. Replies in blue. New text in italics. We provide a clean pdf and tex file, and a copy with new text highlighted.

------------------------------------------------------------------------

a) Please attend to the concerns raised by the reviewers. However, do note the guidance from the Academic Editor. Essentially her/his suggestion is that Rev #1's request about alternative seeding and Rev #2's requests about dysregulation and PD resting state data should be addressed by discussion alone. HOWEVER, Rev #2's query about sensitivity to parcellation and Rev #3's issue about parameters and model fit must be addressed fully.

b) Please address my Data Policy requests below, namely to supply underlying data and to cite its location in the relevant Figure Legends.

------------------------------------------------------------------------

ETHICS STATEMENT:

The Ethics Statements in the submission form and Methods section of your manuscript should match verbatim. Please ensure that any changes are made to both versions.

-- Please include the full name of the IACUC/ethics committee that reviewed and approved the animal care and use protocol/permit/project license. Please also include an approval number if one was obtained.

-- Please include the specific national or international regulations/guidelines to which your animal care and use protocol adhered. Please note that institutional or accreditation organization guidelines (such as AAALAC) do not meet this requirement.

-- Please include information about the form of consent (written/oral) given for research involving human participants. All research involving human participants must have been approved by the authors' Institutional Review Board (IRB) or an equivalent committee, and all clinical investigation must have been conducted according to the principles expressed in the Declaration of Helsinki.

------------------------------------------------------------------------

DATA POLICY:

You may be aware of the PLOS Data Policy, which requires that all data be made available without restriction: <http://journals.plos.org/plosbiology/s/data-availability>. For more information, please also see this editorial: <http://dx.doi.org/10.1371/journal.pbio.1001797>

We note that the model code is in Github and the deformation maps in Neurovault; we also assume that some of the figure panels are generated directly from model outputs or raw-ish data. However, we also require the simpler structured data that is presented in graphs to be made available in one of the following forms:

1) Supplementary files (e.g., excel). Please ensure that all data files are uploaded as 'Supporting Information' and are invariably referred to (in the manuscript, figure legends, and the Description field when uploading your files) using the following format verbatim: S1 Data, S2 Data, etc. Multiple panels of a single or even several figures can be included as multiple sheets in one excel file that is saved using exactly the following convention: S1_Data.xlsx (using an underscore).

2) Deposition in a publicly available repository. Please also provide the accession code or a reviewer link so that we may view your data before publication.

Regardless of the method selected, please ensure that you provide the individual numerical values that underlie the summary data displayed in Figs 2B, 3, 4, 5, 6, 7, S3C, S4, S5, S6 and S9, as they are needed for readers to assess your analysis and to reproduce it. Please also ensure that figure legends in your manuscript include information on where the underlying data can be found.

Please ensure that your Data Statement in the submission system accurately describes where your data can be found

------------------------------------------------------------------------

REVIEWERS' COMMENTS:

Reviewer #1:

Thank you for inviting me to review this manuscript by Zheng and colleagues, in which the authors use an inventive combination of agent-based modeling and multimodal neuroimaging approaches to examine the trans-synaptic spread hypothesis in individuals with Parkinson's disease. The conception and execution of the analyses in the study are exemplary -- indeed, I have no issues to raise with the methodological aspects of the study. My only (admittedly minor) concerns lie with the interpretation of the results. In brief, I consider the lack of detailed brainstem anatomy as a crucial missing piece of the story, and worry that the authors may be slightly over-interpreting their results without due appreciation of this issue. Importantly, I don't think that this fact should impede publication of this important work, but I do maintain that the work would be more useful to the broader community if the issues associated with the missing/hidden nodes in the brainstem was given more

consideration in the manuscript.

I have included below comments which I hope are helpful.

* The authors chose to 'seed' the substantia nigra, as it is commonly identified as a crucial nexus of pathology in Parkinson's disease. While I don't doubt that this is the case, there is a long-standing and influential hypothesis (Braak, 200) that proposes that alpha-synuclein pathology begins in either olfactory cortex or the ventral medulla (likely through vagal afferents from the peripheral nervous system). Would the authors expect to see similar results if they seeded a different region that was more consistent with the known pathological process at play in PD? Or does the inherent uncertainty associated with creating accurate white-matter connectomes in the brainstem limit this line of questioning? Either way, I think that it's important that this issue is argued/justified in the manuscript, lest the reader come away with the opinion that alpha-synuclein pathology somehow manifests in the substantia nigra without affecting other regions/nuclei.

* I worry that few (if any) brainstem structures other than the SN were included in the authors model. Based on the patterns of known pathology, I would expect that connectivity within and between different subregions of the brainstem would have a major influence over patterns of pathological spread within the cortical and subcortical network of the brain.

* Along similar lines, I worry that the focus on the substantia nigra unnecessarily limits the scope of the authors conclusions. What would happen if you seeded another region that is associated with substantial lewy pathology in PD, such as the locus coeruleus, dorsal raphe, pedunculopontine nucleus, to name but a few? Could seeding these regions better explain spread/atrophy associated with some of the other symptoms of the Parkinson's syndrome, such as cognitive impairment, mood disturbance and visual hallucinations?

* On page 10, the authors claim that their results are consistent with the Braak hypothesis, and that the SN is one of the earliest sites affected. I'm not sure that this is entirely accurate -- see Figure 1A of Surmeier, Obeso and Halliday, 2017 (<https://www.nature.com/articles/nrn.2016.178>), which shows that SNc is only affected in Braak Stage III/IV. By that stage, many other regions in the brainstem, forebrain and subcortex are affected, suggesting that the SN is not necessarily that important for disease spread per se (though it may be [and likely is] for motor symptoms].

Incorporating the brainstem

1) We agree that our model would benefit from including the brainstem below the substantia nigra. We also agree that if the Braak model is correct, the disease almost certainly spreads from caudal areas up to the substantia nigra. However, the current state of the art makes including pons and medulla impossible for three reasons:
1. Measuring atrophy from T1 MRI in the brainstem is difficult due to its size (and the greater likelihood of imaging artifacts due e.g. to pulse and respiration). To appropriately model brainstem disease one would want to (a) detect atrophy and (b) pinpoint it accurately to specific nucleii. The brainstem contains small nucleii with relatively few neurons, which however may be affected with significant clinical consequences. For example, Braak’s postmortem specimens typically show considerable pathology of the dorsal motor nucleus of the vagus (Stage I), but absence of involvement of the nearby solitary nucleus (see Figure from Braak et al. *Neurobiol Aging 2003*). It is impossible using current MRI to resolve these structures or accurately measure their volume even though this would be crucial for accurate modeling.

2. Obtaining a brainstem connectome is also currently unfeasible. In addition to being unable to pinpoint the various small nucleii, diffusion imaging cannot disentangle the numerous intricate connections present here. Current work with ex-vivo MRI of brainstem is encouraging and we hope to include these atlases in our future work when they become available.
3. Finally, the Allen Human Brain Atlas is not as rich and precise for gene expression for the lower brainstem i.e. even if we could map atrophy and connectivity in the pons and medulla, it would be challenging to accurately register SNCA and GBA gene expression to the map from patients.

SN as earliest affected site / epicenter

We agree that this section was poorly worded. In particular we now remove this sentence: *“This is consistent with the Braak hypothesis, where the substantia nigra is one of the earliest affected sites in the central nervous system.”* We agree that this was incorrect.

The SN is indeed only affected in Braak stage 3. What we meant is that SN is the epicenter among the brain regions that we can model (i.e. cortex, subcortical structures, midbrain). For the reasons stated above, we cannot include MRI data from pons and medulla.

Also, in the rewritten version we emphasize that we use “epicenter” in the sense of best propagator, rather than first site of outbreak. This definition is commonly used in infection modeling. “Epicenter” is akin to “best propagator” as used by others (e.g. Iturria-Medina et al. 2014). So the SN is not the origin of the disease but, among the regions included in the model, the best at propagating it. This presumably derives from its high levels of alpha-synuclein and widespread connectivity to supratentorial regions.

New text:

*In our model, the substantia nigra has the lowest spread threshold, identifying it as a likely disease epicenter. This is not to say that the substantia nigra is the origin of the disease, or the first affected site: the concept of epicenter as used here is similar to ”best propagator” [10], and identifies the region most likely to trigger an outbreak, rather than the first affected site. According to the Braak hypothesis, the dorsal motor nucleus of the vagus is the initial central nervous system target in PD [31, 32]; however, we could not include structures in the pons and medulla due to difficulty in imaging either atrophy or white matter tracts in the brainstem. Nonetheless, our model is consistent with the substantia nigra acting as a propagator of disease from brainstem to supratentorial areas [19]. We suggest that this may result from its high concentrations of α-synuclein and widespread connections.*

Here we use the “spread threshold” as a measure of the likeliest epicenter. This is the smallest number of infected agents that can be introduced into a region to trigger an outbreak. Its derivation is described here:

*However, in our agent-based higher-order system in which new agents are constantly synthesized and move across regions, the transition threshold can only be determined numerically by scanning across different injected amounts of misfolded α-synuclein to find the point at which the disease no longer extinguishes. More specifically, starting with an injection amount at which the disease does not spread (here we chose 1e-13), we incremented the value by step sizes of 1e-13 until the point where the disease no longer extinguishes, and took this as the spread threshold. This procedure was repeated for every region, yielding 42 regional spread thresholds (Fig 4, red curve).*

(In our previous work (Zeighami et al. eLife 2015) we used a different method to identify the disease epicenter: correlating path length to every node versus atrophy, which also identified the substantia nigra.)

Spread threshold is plotted in Fig 4 for every region.

Seeding the substantia nigra

We did not emphasize an aspect of the S-I-R model clearly enough: the dynamic model reaches an equilibrium such that no matter which region is seeded the final distribution of normal and misfolded alpha-synuclein is the same. Our model can have two fixed points: disease extinction or outbreak (see supplemental Figure S1). This is often the case in S-I-R models where agents are constantly added and removed (such as with births and deaths in a population). So for the purposes of correlating the modeled atrophy to the true atrophy any seed, in this case, will give the same result. The difference is that some areas will be more likely to lead to epidemic versus extinction.

So why use the substantia nigra and why do we claim it is the likeliest epicenter? This is addressed above and in the Results section titled “Identifying the disease epicenter”. Here the measure of epicenter is “most likely to initiate disease outbreak”, as determined by the spread threshold, or the minimally-required injection amount of misfolded α-synuclein to initiate an outbreak. As shown in Fig. 4 the substantia nigra has the lowest spread threshold (as well as the highest density of α-synuclein). Note this doesn’t mean the disease starts in the substantia nigra, but that it is the likeliest site to propagate the infection. Finally, as stated above, we could not seed a site caudal to the substantia nigra due to unavailability of a good brainstem connectivity map.

Reviewer #2:

In this manuscript, Zheng and colleagues report on an agent-based model, incorporating multiple aspects PD pathophysiology, aiming for predicting the brain regions first affected by neuronal loss. The approach is highly innovative and novel, as for the first time, multiple divergent parameters are taken into account, which were previously not incorporated into one model. The authors integrate structural connectivity signatures, gene expression data as well as resting state functional connectivity. The model seems to be able to replicate the typical course of PD disease development in terms of regions first affected by neuronal loss, and, identifies the substantia nigra as regions with highest vulnerability. While I cannot judge the merits of the model in mathematical terms, it seems to overcome limitations of previous simpler models, such as diffusion models, and seems to be ideally suited to simulate and test the relatively novel hypothesis of a seed-based disease course,

spreading throughout the network. Notably, they attribute at least theoretical evidence for the hub-neuron hypothesis. The manuscript is well written, and of high interest particularly outside of the computation neuroscience community. However, it falls short on three essential aspects:

1.	The authors focus on the endpoint of local and global disease development: neuronal loss. All of the benchmarks the authors present, are late-stage human PD data, in which regional atrophy is apparent. While this might still be meaningful, it does not at all reflect the current view of disease development. We know now, that much prior to neuronal loss, the network becomes dysregulated, and these dysregulations often entail local hyperactivity. This hyperactivity itself then might lead to subsequent neuronal loss, and reflect back on the molecular disease entity, please see e.g. Iaccarino et al,, Nature 2016, and Zott et al, Ann rev Neur 2018 . These dysregulated network components are not necessarily identical to those networks which degenerate first. The authors need to extend their approach to incorporate these current developments. Ultimately, it is of interest to identify the dysregulated hubs, prior to irrevocable neurodegeneration.

Focus on late-stage PD

The patients included here are early stage PD (Hoehn and Yahr stages 1-2, unmedicated, and diagnosed within 7 months of MR imaging), and demonstrate atrophy in regions that go up to Braak Stage IV (described in our previous work: Zeighami et al ELife 2015). This somewhat contradicts the clinical viewpoint that atrophy is a late-stage phenomenon in PD - but this is a notion that predates the availability of big data like PPMI. Indeed, there are also reports of atrophy in prodromal PD patients with REM Behavior Disorder (RBD) but no parkinsonism (see Rahayel et al. Sleep 2019 <https://doi.org/10.1093/sleep/zsz062>).

We have recently applied the DBM analysis to RBD patients with normal motor exams and found that those who later went on to develop parkinsonism had detectable brain atrophy in a similar but less extensive pattern to the early PD patients in PPMI (this will be submitted as a preprint very soon). In sum, early and prodromal PD patients already show brain atrophy.

Nonetheless, we agree that neuronal atrophy is likely not the earliest dysfunction in PD: it’s likely that the disease process starts many years before motor symptoms appear, and may involve neuronal signaling abnormalities prior to actual neurodegeneration. However, it is not feasible at this time to study a large cohort of prodromal patients with measures of neuronal signaling such as fMRI: the data are not available. Note that we are currently working to fit our agent-based model to data from prodromal individuals with RBD (our own cohort and some of the patients in PPMI).

Also it is important to point out that atrophy detected by MRI may not indicate neuronal loss per se. Here what we show is that the predicted spatial pattern of synuclein accumulation correlates with brain volume loss measured by T1 MRI. Atrophy may well represent loss of neurons, but could also represent loss of synapses or dendrites. Therefore we have now mostly amended references to “neuronal death” in the manuscript, replacing it with “tissue loss” or “synaptic loss” as appropriate. We also added a short sentence on what DBM tissue contraction might represent biologically. We cite a review of the pathology in PD by Halliday et al. suggesting that neuronal loss in many regions may remain modest even in late stage PD (i.e. abnormal synueclein accumulation can lead to synaptic loss with preservation of neurons). We also note that in normal human senescence, there is no cortical neuronal loss but reduction in the number of synapses and dendritic spines (Hof and Morrisson, TINS 2004), and that this is accompanied by atrophy detected by MRI. New text:

*“However, atrophy measured with DBM does not necessarily reflect death of neurons. Indeed, postmortem studies in PD demonstrate a dissociation between alpha-synuclein pathology and neuronal loss, which is prominent in some areas (e.g. substantia nigra), but virtually absent in others (cortex, amygdala) [72]. This is similar to normal senescence, where there is widespread tissue atrophy in cortex despite preservation of neuronal numbers [73]. Thus, it is possible that tissue atrophy in PD may also reflect loss of dendritic arbors and spines without loss of neurons.”*

2.	Related to my first comment, the authors use a resting state data from healthy controls. How can the authors relate their model to a functional connectome, which does not reflect the connectome of a PD patients? They should incorporate the resting state signatures of different stages of PD disease development.

We agree in principle, however it is not clear what are the best imaging data to use for connectivity in this case (i.e. normal connectivity or that derived from patients). Our approach is to model protein propagation that occurred up to the current timepoint and led to the observed atrophy. Therefore, a normal connectome might be better suited than one based on patients with disease. The normal connectome would reflect the pathways used for propagation to first set the degenerative process in motion. Indeed, several other groups who modeled Alzheimer’s Disease used normal connectomes (e.g. Zhou: <https://doi.org/10.1016/j.neuron.2012.03.004>; Raj: <https://doi.org/10.1016/j.neuron.2011.12.040>; Iturria-Medina: <https://doi.org/10.1371/journal.pcbi.1003956>).

Modeling follow-up atrophy in the PPMI dataset, which we are currently doing, could gain from using connectivity derived from the same patients. We will investigate the use of patient-derived DTI data for this purpose in the future.

On the other hand, resting state fMRI data in PD are unfortunately relatively few. Only a tiny subset of participants underwent this procedure at time-point #1 in PPMI (fewer than 20 in the patient and control groups).

We add the following text to the Methodological Considerations section:

*Also, the structural and functional connectomes used here were derived from healthy individuals, as typically done [9, 10, 19, 60]. However, disease-related alterations to cell integrity should eventually affect network function. Connectomics data in PD are starting to become available and it will be interesting to incorporate these time-varying effects into our agent-based model.*

3.	They authors used a parcellation approach, which certainly makes sense. However, could the authors comment on the stability of their model, if the number of parcellations is increased or decreased?

We agree that it is important to test the stability of the model to variations in the number of parcels. We only tested finer parcellations. As we are using correlation analysis to assess model fit, care must be taken in choosing the sample size (i.e., the number of parcels) to achieve a high statistical power. Under a medium effect size (e.g., Pearson’s Correlation Coefficient r=0.5), to have a statistical power of 0.9 at significance level 0.05, the desired sample size is no less than 38. Also, generating a coarser parcellation than 42 regions per hemisphere would imply combining parcels that are functionally and structurally different. Therefore, we tested model stability by increasing the number of parcels, although the 42-region parcellation likely achieves a good balance between signal-to-noise ratio and structural detail.

We chose a hierarchical parcellation scheme that breaks down the 68 DKT cortical regions into smaller parcels (Cammoun et al., 2011), resulting in a partition of 112 regions, and by further breaking it down, resulting in a partition of 217 regions. Combining these cortical partitions with the subcortical segmentations (Fischl et al., 2002) and the substantia nigra (Keuken et al., 2014), we finally constructed 65-region and 119-regions left hemisphere parcellations to feed into the model. Deterministic tractography was conducted on the two new parcellations in the native space for each of the 1027 subjects, as documented in the main manuscript, yielding two group connectivity matrices respectively for the two new parcellations (binary density 40%). This is added as Fig S6:

Fig 2. Model fit at 65-region and 119-region resolution. The 42 regions used in the main manuscript were hierarchically partitioned into 65 regions and further 119 regions. Simulations were conducted on these two finer resolutions, and yielded comparable results to the model fit at 42-region resolution. A: Spearman’s correlation (blue curve) and Pearson’s correlation (red curve) along time course using the 65-region parcellation. Black dot: peak position of the correlation coefficients. B: The model has more predictive power than its constituent factors (as assessed by Spearman’s correlation) at 65-region parcellation. C: Spearman’s correlation (blue curve) and Pearson’s correlation (red curve) along time course using 119-region parcellation. Black dot: peak position of the correlation coefficients. D: The model has more predictive power than its constituent factors (as assessed by Spearman’s correlation) at 119-region parcellation.

As expected, increasing the number of parcels reduces the correlations (or effect size, both Pearson’s and Spearman’s) between simulated atrophy and the real atrophy. This is likely due to lower signal-to-noise in smaller brain parcels. However, at both resolutions the correlations are still highly significant. At 65-region resolution, the simulated atrophy bears Pearson’s r=0.43 (p<0.001) and Spearman’s r=0.41(p<0.001) with the real atrophy, better than GBA or SNCA expression alone (GBA: Pearson’s r=-0.25, Spearman’s r=-0.16; SNCA: Pearson’s r=-0.23, Spearman’s r=-0.21) and network metrics (node strength: Pearson’s r=0.41, Spearman’s r=0.34; node degree: Pearson’s r=0.14, Spearman’s r=0.06; eigenvector centrality: Pearson’s r=0.28, Spearman’s r=0.18); and the same is for 119-region resolution (model fit: Pearson’s r=0.34, p<0.001, Spearman’s r=0.31, p<0.001; GBA: Pearson’s r=-0.23, Spearman’s r=-0.09; SNCA: Pearson’s r=-0.23, Spearman’s r=-0.15; node degree: Pearson’s r=0.09, Spearman’s r=0.03; node strength: Pearson’s r=0.34, Spearman’s r=0.29; eigenvector centrality: Pearson’s r=0.29, Spearman’s r=0.21), suggesting the model is stable across different parcellation schemes. All the other parameters used in the model are as documented in the main manuscript (network density ~40%, $\rho$=0.5, $k_{1}/k_{2}$=1).

Reviewer #3:

Summary:

Authors develop an agent-based epidemic (SIR) model to represent the spread of pathological proteins in neurodegenerative diseases.

Their approach integrates structural connectivity, functional connectivity and gene expression, to predict sequential volume loss due to neurodegeneration.

1. Their model concludes implicates the substantia nigra as the “disease” epicenter.

2. Their model concludes that SNCA and GBA transcription influence alpha-synuclein concentration and local regional vulnerability.

3. Their model concludes that functional co-activation further amplifies the course set by connectome architecture and gene expression.

Comments:

1. The choice of an epidemic model seems well-motivated in the Intro.

2. It is evident that this work represents a thorough and thoughtful effort in bringing together several important datasets. All datasets and code have been made available to the community, and the authors' insights are a nice jumping-off point for continued exploration (by them, or others).

Suggestions:

Ultimately, the model fit is not overly convincing, in particular given the number of parameters tuned - peaking at correlation 0.6. That said, I personally do not feel that this fact detracts from the importance or publish-ability of this work. Authors have made explicit how they selected their parameters, and follow-up work can explore variations thereof. However, I think authors could include a brief discussion of the "goodness" of this result, in their view, given broader context.

We thank the reviewer for evaluating the manuscript.

Certainly the fit is not perfect - the reasons are probably that tissue loss measured with T1 MRI is an imperfect measure of disease, and that diffusion weighted imaging does not provide an exact measure of connections. In addition the spatial resolution of MRI will always limit measurement accuracy. We note however that our approach is perhaps more rigorous than much of the AD literature where spatial correlations are often not even provided, and null models either absent or less extensive!

Moreover, the model fits obtained here are of the same order (pearson correlations 0.5 - 0.7) as those recently described by Henderson et al. (*Nature Neuroscience* 22, 1248–1257; 2019) in mice. One might expect far better fits in the mouse model since in that study there was direct high-resolution observation of synuclein pathology with microscopy, and a far better connectome in mouse (based on tract tracing from the Allen Institute). It is comforting to see that the human model achieves similar fits despite the greater accuracy of the mouse dataset. Perhaps the advantage of our model is the use of an agent-based framework over the simple diffusion model used by Henderson et al.

pages

The two main reasons we take the fit obtained here as a good test of our model however are (1) that it is degraded when we randomize either the connectome or the gene expression values, meaning that atrophy depends on both of these biological features and (2) that the full model with propagating agents provides a better fit than the other commonly used network metrics (degree, eigencentrality) or simple gene expression (SNCA and GBA), supporting the theory that it is both connections and local features that shape disease expression. Also, we generate null models by rewiring the connectome and changing the spatial location of nodes, and by shuffling gene expression

COMMENTS FROM THE ACADEMIC EDITOR:

My take is that both R1 and R2 are essentially requiring relatively minor revisions.

I think that it is clear that R1 demands that the authors requires alternative seeding (and therefore brain stem anatomy) should be taken on board as a discussion issue. This discussion will lay the basis of a future study.

We now explain that existing atlases and methods do not allow for integration of brainstem pathology and connectivity into the model, but that there will soon be such atlases that could be combined with other data i.e. postmortem data in humans; novel MRI approaches that use diffusion MRI and neuromelanin sequences to detect pathology in brainstem.

My interpretation of R2 is much along the same lines. In R2's first point, addressing dysregulation (rather than neuron loss) would be much closer to addressing the onset of the disease and therefore the factors that are more directly involved in the pathological spreading (which is the long-term aim of the study).

Likewise R2's second point is coming back to disease onset by asking that the authors should use resting state data from PD patients (preferably in early onset of the disease). These are two very valid points that I think will constitute a future refinement of the model that will find itself in a future study.

We address this as well. Resting state fMRI data in PD patients simply do not exist in large enough quantity at present (N=20 in PPMI). We and others are currently collecting this data for future tests of the agent based model.

HOWEVER, varying the parcellation (R2 point 3) directly applies to the present study and needs to be addressed. As does R3's issue of choice of parameter and goodness of fit of the model.

In response to R3, we have repeated the analysis with two finer parcellations. We also address the goodness of fit issue. Our understanding is that R3 was OK with our justification for the chosen model parameters. We tested these by generating several null models (rewiring the connections; changing the spatial positions of the nodes; and shuffling gene expressions). In Figures S8 and S9 moreover we tested the robustness of the model to different parameters.

In summary I feel that should the paper be revised by including the additional analysis suggested in R1 and R2 it will be come overtly cumbersome and counter productive. However, a full discussion of the issues raised by R1 and R2 will greatly help elucidate the perspectives of the study.

We have added new text for discussion of their major points to the manuscript. We also added a panel to Fig 7 to clarify how we use functional connectivity in the model.Finally, we have made all our data and code available on github, added an author significance statement, and amended the formatting to fit PLoS guidelines.
